# Sustainable aggregate production planning in the chemical process industry - A benchmark problem and dataset

**DOI:** 10.1016/j.dib.2018.03.064

**Published:** 2018-03-30

**Authors:** Marcus Brandenburg, Gerd J. Hahn

**Affiliations:** a Flensburg University of Applied Sciences, School of Business, Kanzleistraße 91–93, 24943 Flensburg, Germany; b University of Kassel, Chair of Supply Chain Management, Kleine Rosenstraße 1–3, 34117 Kassel, Germany; c German Graduate School of Management and Law, Professorship of Operations Management and Process Innovation, Bildungscampus 2, 74076 Heilbronn, Germany

## Abstract

Process industries typically involve complex manufacturing operations and thus require adequate decision support for aggregate production planning (APP). The need for powerful and efficient approaches to solve complex APP problems persists. Problem-specific solution approaches are advantageous compared to standardized approaches that are designed to provide basic decision support for a broad range of planning problems but inadequate to optimize under consideration of specific settings. This in turn calls for methods to compare different approaches regarding their computational performance and solution quality. In this paper, we present a benchmarking problem for APP in the chemical process industry. The presented problem focuses on (i) sustainable operations planning involving multiple alternative production modes/routings with specific production-related carbon emission and the social dimension of varying operating rates and (ii) integrated campaign planning with production mix/volume on the operational level. The mutual trade-offs between economic, environmental and social factors can be considered as externalized factors (production-related carbon emission and overtime working hours) as well as internalized ones (resulting costs). We provide data for all problem parameters in addition to a detailed verbal problem statement. We refer to Hahn and Brandenburg [1] for a first numerical analysis based on and for future research perspectives arising from this benchmarking problem.

**Specifications table**

**Table:** 

Subject area	Operations Research
More specific subject area	Aggregate Production Planning
Type of data	Table, Figure
How data was acquired	A benchmark problem introduced by Papageorgiou and Pantelides [2] is complemented by other scientific sources
Data format	Raw
Experimental factors	Data can be aggregated for hierarchical aggregate production planning
Experimental features	Data contains a benchmark problem for hierarchical aggregate production planning in the chemical process industry
Data source location	Not applicable
Data accessibility	Data is within this article

**Value of the data**

• The dataset at hand serves as a benchmark problem for hierarchical aggregate production planning in the chemical process industry. For this purpose, the data is represented by a state-task-network and complemented by further information for mid-term planning (esp. demand quantities and aggregate cost parameters).
• The dataset provides parameters that capture the stochasticity of the manufacturing system (e.g. equipment availability, variation of setup and processing times). Consequently, this dataset can be used for stochastic analyses of a manufacturing system.
• Furthermore, the dataset includes information about energy cost and carbon emissions as well as information on social factors, in particular overtime, that allow for an analysis with respect to sustainability issues.

## 1 Data

The benchmark problem presented in this paper arises from aggregate production planning (APP) in the chemical process industry. To avoid problems regarding data confidentiality and to ensure scientific rigor in the formulation of the benchmarking problem, we have combined published data from publicly available sources, in particular scientific manuscripts.

The production system and processes of the benchmark problem are based on a case example of a chemicals manufacturer. This case example has been used to numerically analyze procedures for short-term campaign scheduling over a planning horizon of about ten weeks (see, e.g., [2], [6], [7]). However, the manufacturing system and processes of the case example are adequate to numerically illustrate and analyze mid-term APP approaches.

Numerous characteristics of planning and scheduling in the process industry (see, e.g., [3], [4], [5] for a taxonomy) are reflected in the benchmark problem. Following the classification introduced by Méndez et al. [5], the benchmark problem comprises batch processes with variable sizes and fixed, unit-independent processing times. These processes are operated on multi-purpose production units with full connectivity in multi-stage production with an instantaneous and, thus negligible, material transfer. Regarding inventory storage, all kinds of quantity- and time-based constraints apply. Non-working periods are caused by shifts and can be resolved by additional overtime. Due dates of multiple product demands are distributed across a fixed planning horizon of one year. Stochasticity arises from variations of setup and processing times and furthermore equipment unavailability.

Realistic magnitudes of additional numerical parameters, in particular financial figures (see [8], [9]), operational figures (see [10], [11], [12], [13], [14]) and environmental figures (see [15]), are also derived from related literature. Unit-specific differences in carbon emissions [15] represent the environmental dimension of sustainability. Overtime costs reflect workers' preferences to work during regular shifts [16] and, thus, represent the social sustainability dimension. Information on shift patterns or non-working periods during weekends in process industry is taken from Méndez et al. [5], Northrup et al. [17], Shaik et al. [18] and Eberle et al. [19]. Demand seasonality is obtained from Jones et al. [13] and Shah [14]. Ranges of these problem instance parameters are listed in Subsection 2.3 *Problem instance* data.

## 2 Experimental design, materials and methods

### 2.1 Base data

The production system manufactures three finished products (A, B, C) from three raw materials and ten intermediate products. As illustrated in Table 1, the processing equipment consists of eight multi-purpose units (u1, …, u8) with different capacities on which 13 processes (P1.A, …, P5.A, P1.B, …, P3.B, P1.C, …, P5.C) are operated. A total of 23 different tasks result from the process-unit-assignment. Each task represents a unique production mode. Process durations depend on the particular process and on the chosen processing unit.

**Table 1:** Details of processing equipment based on Papageorgiou and Pantelides [2].

	**Size**	**Feasibility**		
**Unit**	in tons	Finished product A	Finished product B	Finished product C
u1	40.0	P1.A, P3.A	–	P1.C
u2	10.0	–	–	P2.C, P4.C, P5.C
u3	10.0	–	–	P2.C, P4.C, P5.C
u4	30.0	–	P1.B	P3.C
u5	15.0	P5.A	P1.B, P3.B	P3.C
u6	40.0	P2.A, P4.A	P2.B	–
u7	15.0	P3.A	P3.B	P5.C
u8	50.0	P4.A	–	P3.C

Inventory storage is constrained by quantity (unlimited (UIS), finite (FIS) or no inventory storage (NIS)) and time (unlimited (UW), finite (FW) or zero wait (ZW) storage durations).^1^ Some buffer capacities are limited and zero wait conditions apply for some processes. As a consequence, the tasks can be aggregated to in total five different stages, each of them consisting of two or three processes. These stages are separated by different raw materials, intermediate and finished products which can be stored in substantial quantities. In each stage, different process-unit assignments are feasible and lead to in total 28 different production routings.^2^

**Fig. 1:**
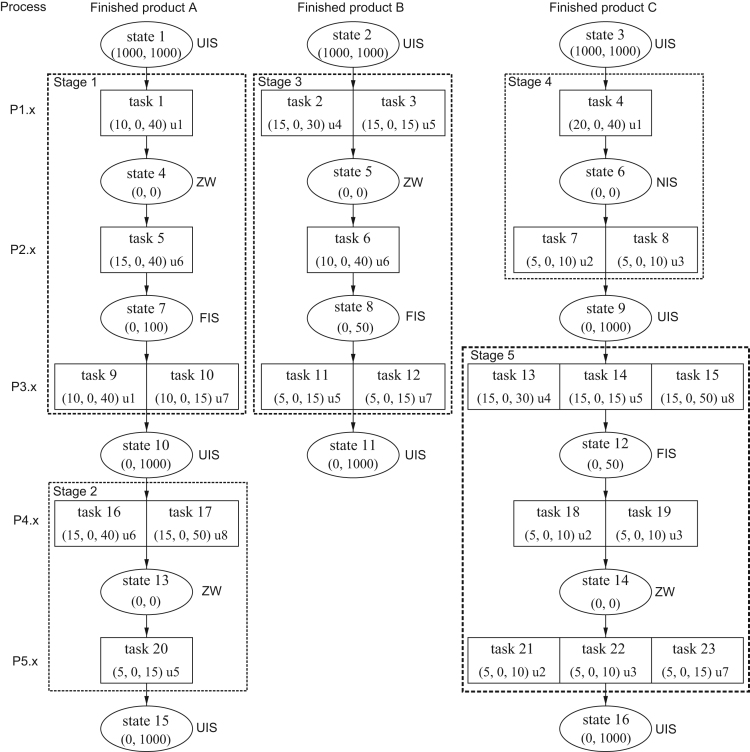
State-task-network of the chemical processes based on Papageorgiou and Pantelides [2], p. 30, and Burkardt and Hatzl [7], p. 1178, and developed further by Hahn and Brandenburg [1].

Fig. 1 illustrates the chemical processes of the case example as a state-task-network (STN), a concept which has been introduced by Kondili et al. [21]. In this STN, ovals represent states and the numbers in parentheses inform about initial and maximum inventory levels. Furthermore, rectangles represent tasks for the conversion of states, contain data for process duration, minimum and maximum batch size (numbers in parentheses) and inform about the production unit (u1, …, u8) on which the task is operated. Dotted lines encircle the tasks that can be grouped to the same stage if storage constraints (FIS, NIS or ZW) between subsequent processes apply.

### 2.2 Planning parameters

The available capacities have to be balanced with customer demands. Minimum batch sizes and maximum campaign sizes have to be considered. The planning horizon of 12 months covers a calendar year and a full seasonal cycle. Production equipment is operated over five days with two shifts of eight hours per day which results in a weekly base capacity of 80 hours. This base capacity can be extended to as much as 168 hours per week by overtime at cost of 640 currency units (CU) per hour.

### 2.3 Problem instance data

In addition to data provided by Papageorgiou and Pantelides [2], the following problem instance parameters are relevant for the benchmarking problem presented.

Base demands per month vary between 80 and 240 t. Seasonality is modeled using a trigonometric function with an amplitude of 20% and a peak in the middle of the planning period. Inventory holding costs are determined as 12% p.a. of cost of goods sold. Corresponding backlog cost rates are derived according to a fill rate of 98.5%. The initial inventory of intermediate and finished production stages corresponds to 1.2–1.4 as well as 1.3–1.8 times the base demand per month which reflects average lead times. There is no initial and final backlog; inventory capacity at each stage is limited to 1000 t. Initial WIP inventories equal the base demand of one period. Detailed values are provided in Table 2.

**Table 2:** Demand and cost parameter per production stage.

Stage	Monthly Base demand	Initial Inventory	Inventory cost per ton and month [CU]	Backlog cost per ton and month [CU]

1	200	280	8.87	591.0
2	80	140	12.85	856.4
3	240	320	10.37	691.4
4	200	240	7.66	510.3
5	80	120	13.70	913.3

Each processing unit is available at 100%. Setup processes have a duration of 8 hours (1 shift). The squared coefficient of variation of processing time is 0.2 for setup processes and 0.4 for conversion processes. Carbon emissions per ton of production depend on the specific processing unit and vary between 85% and 120% of the emission level of processing unit u1 (see Table 3).

**Table 3:** Energy/CO2 factor for each processing unit.

Processing unit	u1	u2	u3	u4	u5	u6	u7	u8
Energy/CO2 factor per ton and hour	1.00	0.90	1.10	0.85	0.95	0.85	1.20	1.05

A campaign on a specific production routing comprises at least one batch ranging in the size of 30 to 50 t. The routing-specific maximum campaign size equals 10 batches. The routing sequence and corresponding cost parameters are provided in Table 4.

**Table 4:** Routing sequences, batch sizes and cost parameters per production routing.

Stage	Product routing	Sequence	Batch size [tons]	Energy cost per ton [CU]	Production cost per ton [CU]	WIP cost per ton and month [CU]

1	1	u1;u6;u1	40	85	175	3.99
1	2	u1;u6;u7	40	95	275	4.88
2	3	u6;u5	40	75	150	10.94
2	4	u8;u5	45	60	133	10.78
3	5	u4;u6;u5	30	65	233	4.87
3	6	u4;u6;u7	30	55	233	4.97
3	7	u5;u6;u5	30	110	333	5.40
3	8	u5;u6;u7	30	100	333	5.50
4	9	u1;u2	40	60	200	3.80
4	10	u1;u3	40	60	200	3.86
5	11	u4;u2;u2	30	45	300	10.73
5	12	u4;u2;u3	30	45	300	10.80
5	13	u4;u2;u7	30	45	300	10.80
5	14	u4;u3;u2	30	45	300	10.80
5	15	u4;u3;u3	30	45	300	10.86
5	16	u4;u3;u7	30	45	300	10.86
5	17	u5;u2;u2	30	60	300	10.62
5	18	u5;u2;u3	30	60	300	10.68
5	19	u5;u2;u7	30	60	300	10.68
5	20	u5;u3;u2	30	60	300	10.68
5	21	u5;u3;u3	30	60	300	10.75
5	22	u5;u3;u7	30	60	300	10.75
5	23	u8;u2;u2	50	80	260	10.52
5	24	u8;u2;u3	50	80	260	10.58
5	25	u8;u2;u7	45	70	244	10.40
5	26	u8;u3;u2	50	80	260	10.58
5	27	u8;u3;u3	50	80	260	10.65
5	28	u8;u3;u7	45	70	244	10.47

### 2.4 Data modeling and mapping

The process details of the STN may need to be aggregated in order to reduce granularity and complexity to an appropriate level. For this purpose, an algorithmic preprocessing procedure can be executed to determine alternative production routings and resulting capacity requirements for each stage. The three steps of the preprocessing procedure are executed as follows:

**Step 1** The processes are aggregated to stages if storage constraints (FIS, NIS or ZW) between subsequent processes apply and all possible routings are determined for each stage. Example: Stage 1 comprises two different routings (u1-u6-u1 and u1-u6-u7) to operate the three processes (P1.A-P2.A-P3.A). In contrast, stage 3 comprises four different routings (u4-u6-u5, u4-u6-u7, u5-u6-u5 and u5-u6-u7) to operate the three processes (P1.B-P2.B-P3.B).
**Step 2** The capacity requirement is determined for each stage *S* and each routing *m*: The number *n*∈ℕ and size *κ*_*v*_∈ℝ_0_^+^ of batches for each task *v* with maximum batch size $k_{v}^{max}$ is determined that minimizes the total unused capacity Σ_*ν*∈S_ ($k_{v}^{max}$ – *κ*_*v*_) while ensuring that at least one task is executed at most once and that the material flow constraints, i.e. the mass balances given by (n_*v*_ – 1) *·* $k_{v}^{max}$ + *κ*_*v*_ = (n_*w*_ – 1) *·* $k_{w}^{max}$ + *κ*_*w*_ for each task *v* with predecessor task *w*, are not violated. Example: For stage 1, the routing u1-u6-u1 can be chosen without resulting capacity loss (each task is executed exactly once with maximum batch size 40) while routing u1-u6-u7 would leave 5 capacity units unused (tasks 1 and 5 are each executed once with maximum batch size and task 10 is executed twice with maximum batch size 15 and once with batch size 10).
**Step 3** For each stage and each routing, the capacity requirement is calculated. For each unit of the routing and each task *v* that is operated on this unit, the duration of v is multiplied with the number n_*v*_ calculated in step 2. Example: For stage 1, routing u1-u6-u1 requires 20 h of unit u1 and 15 h of unit u6 to produce an output of 40 t while routing u1-u6-u7 requires 10 hours of u1, 15 hours of u6 and 30 h of u7 to produce 40 t.

This preprocessing step ensures that production batches within one stage are synchronized regarding input and output quantities while the time-based synchronization remains a detailed scheduling task. Therefore, intermediate products within one stage are implicitly considered but not modeled explicitly or reflected by mass balances. Hence, the set of products comprises only the states 11, 15, and 16 as finished products and the states 9 and 10 as intermediate products.

## Footnotes

1 See Neumann et al. [20] for a categorization of inventory storage constraints.

2 A routing is defined for a particular stage and determines which unit is chosen to operate each process of this stage. Example: Stage 1 comprises two different routings (u1-u6-u1 and u1-u6-u7) to operate the three processes (P1.A-P2.A-P3.A).
