# Supplementary material for: Sustainable aggregate production planning in the chemical process industry - A benchmark problem and dataset
Source: Data Brief. 2018 Mar 30;18:961–7. doi: 10.1016/j.dib.2018.03.064 (PMC5996297; doi:10.1016/j.dib.2018.03.064)
Supplement: Supplementary file 1 — Supplementary material [file mmc1.pdf]

AUTHOR DECLARATION

Title: *Sustainable aggregate production planning in the chemical process industry - A benchmark problem and dataset*
Authors: Marcus Brandenburg [a,*], Gerd J. Hahn [b]
Affiliations: [a] Flensburg University of Applied Sciences, School of Business, Kanzleistraße 91-93, 24943 Flensburg, Germany – University of Kassel, Department of Supply Chain Management, Kleine Rosenstraße 1-3, 34117 Kassel, Germany [b] German Graduate School of Management & Law, Professorship of Operations Management and Process Innovation, Bildungscampus 2, 74076 Heilbronn, Germany * Corresponding author
Contact email: marcus.brandenburg@hs-flensburg.de (Marcus Brandenburg), gerd.hahn@ggs.de (Gerd J. Hahn)

We wish to confirm that there are no known conflicts of interest associated with this publication and there has been no significant financial support for this work that could have influenced its outcome.

We confirm that the manuscript has been read and approved by all named authors and that there are no other persons who satisfied the criteria for authorship but are not listed. We further confirm that the order of authors listed in the manuscript has been approved by all of us.
